# The SH-SY5Y cell line in Parkinson’s disease research: a systematic review

**DOI:** 10.1186/s13024-017-0149-0

**Published:** 2017-01-24

**Authors:** Helena Xicoy, Bé Wieringa, Gerard J.M. Martens

**Affiliations:** 1 Department of Cell Biology, Radboud Institute for Molecular Life Sciences (RIMLS), Radboudumc, Nijmegen, The Netherlands; 2 Department of Molecular Animal Physiology, Donders Institute for Brain, Cognition and Behaviour, Radboud University, Nijmegen, The Netherlands

**Keywords:** Cell culture conditions, Cellular differentiation, Cellular model, Dopaminergic neuron, Neuroblastoma, Parkinson’s disease, SH-SY5Y cell line

## Abstract

Parkinson’s disease (PD) is a devastating and highly prevalent neurodegenerative disease for which only symptomatic treatment is available. In order to develop a truly effective disease-modifying therapy, improvement of our current understanding of the molecular and cellular mechanisms underlying PD pathogenesis and progression is crucial. For this purpose, standardization of research protocols and disease models is necessary. As human dopaminergic neurons, the cells mainly affected in PD, are difficult to obtain and maintain as primary cells, current PD research is mostly performed with permanently established neuronal cell models, in particular the neuroblastoma SH-SY5Y lineage. This cell line is frequently chosen because of its human origin, catecholaminergic (though not strictly dopaminergic) neuronal properties, and ease of maintenance. However, there is no consensus on many fundamental aspects that are associated with its use, such as the effects of culture media composition and of variations in differentiation protocols. Here we present the outcome of a systematic review of scientific articles that have used SH-SY5Y cells to explore PD. We describe the cell source, culture conditions, differentiation protocols, methods/approaches used to mimic PD and the preclinical validation of the SH-SY5Y findings by employing alternative cellular and animal models. Thus, this overview may help to standardize the use of the SH-SY5Y cell line in PD research and serve as a future user’s guide.

**Electronic supplementary material:**

The online version of this article (doi:10.1186/s13024-017-0149-0) contains supplementary material, which is available to authorized users.

## Background

Parkinson’s disease (PD) is the second most common neurodegenerative disease with a predicted prevalence of 9 million people worldwide by 2030 [1, 2]. PD has a high socioeconomic burden since it is slowly progressive and disease-modifying treatments are not available. PD presents with motor and non-motor symptoms [3, 4] that worsen with advancing age, leading to a need for assistance with all daily activities. Disease manifestation is characterized by the presence of Lewy bodies (abnormal protein aggregates containing α-synuclein), death of dopaminergic (DAergic) neurons in the substantia nigra (SN) projecting to the striatum, and microgliosis (accumulation of activated microglial cells) [5]. However, the molecular mechanisms underlying all these disease features are unknown, hampering the development of effective treatment. In order to understand the pathophysiological mechanisms underlying PD and develop disease-modifying therapies, it is necessary to have adequate models for in vitro and in vivo studies.

An in vitro model widely used in PD research is the neuroblastoma SH-SY5Y cell line. This line is a subline of the SK-N-SH cell line, which was established in culture in 1970 from a bone marrow biopsy of a metastatic neuroblastoma of a 4-year-old female and has undergone three rounds of clonal selection [6]. The initial characterization of the SH-SY5Y cell line showed moderate activity of dopamine-β-hydroxylase and negligible levels of choline acetyl-transferase, acetylcholinesterase and butyryl-cholinesterase [6], basal noradrenaline (NA) release [7] and tyrosine hydroxylase activity [8]. Tyrosine hydroxylase is the rate-limiting enzyme of the catecholamine synthesis pathway and converts tyrosine to L-dopa [9], the precursor of dopamine (DA), which is converted to NA by dopamine-β-hydroxylase [10]. Therefore, the SH-SY5Y cell line may display a catecholaminergic phenotype since it has the machinery to synthesize both DA and NA. Although these properties do not classify SH-SY5Y cells as purely DAergic, this cell line has been widely used as a model for PD. The SH-SY5Y cell line displays a number of genetic aberrations due to its cancerous origin, but most genes and pathways dysregulated in PD pathogenesis are intact [11]. However, the use of an oncogenically transformed cell line with catecholaminergic rather than exclusively DAergic properties remains a controversial issue in the PD field.

The purpose of this systematic review is to provide an overview of the value and use of the SH-SY5Y line as a cell model for PD. We describe in detail the culture conditions, the methodology used to provoke the onset of differentiation, the techniques to mimic typical pathogenic PD features and the alternative models used to validate the findings. Moreover, the limitations of the use of the cell line will be discussed.

## Main text

For our review, we conducted a standard systematic literature search in PubMed which included the terms “Parkinson”, “Parkinson’s”, or “Parkinson’s disease” and “neuroblastoma”, “SH-SY5Y”, “SHSY5Y” or “SHSY-5Y”. The application of these search terms aimed to cover most of the literature regarding the use of the SH-SY5Y cell line in PD research, missing only those studies in which the above-mentioned terms are only present in the main text but not in the title, abstract or MeSH terms. The search performed the 22^nd^ of November 2016 retrieved 1489 articles, of which 962 were original, accessible and PD-specific papers and thus were included in the analysis. The exclusion criteria for the remaining 527 articles were: (i) written in a language different from English, (ii) represents a review, (iii) not specific for PD, (iv) Parkinson as an author, (v) the cell line was mentioned but used in previous studies, and (vi) use of a neuroblastoma cell line different from SH-SY5Y (see Additional file 1, for full list and exclusion details).

### Cell source and culture conditions

**Fig. 1:**
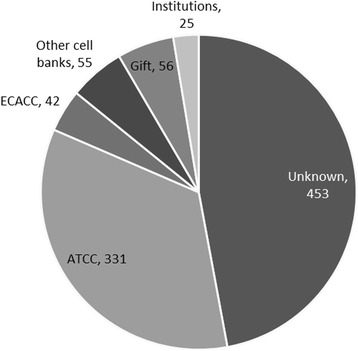
Sources from which researchers obtained the SH-SY5Y cell line. Number of articles using a particular source is indicated. ATCC: American Type Culture Collection; EACC: European Collection of Authenticated Cell Cultures; other cell banks includes all of them, except for ATCC and ECACC

The most-reported source for access to the SH-SY5Y cell line is the American Type Culture Collection (ATCC, CRL-2266, deposited by JL Biedler). Other sources concern retrieval from other cell banks, such as the European Collection of Authenticated Cell Cultures (ECACC, Catalog number: 94030304, deposited by PFT Vaughan) or the German collection of Microorganisms and Cell Cultures (DSMZ, ACC 209). Cells were also obtained through gifts from colleague scientists (Fig. 1). However, in 455 out of 962 publications the cell origin was not specified.

Recommendations regarding the composition of the growth medium for propagation of SH-SY5Y cells vary among the various cell line distributors: ATCC recommends MEM/F12 supplemented with 10% fetal bovine serum, ECCAC recommends MEM/F12 with 2 mM glutamine, 1% non-essential amino acids and 15% fetal bovine serum, and DSMZ recommends MEM plus 15–20% fetal bovine serum. In the actual protocols employed in the PD-related publications, DMEM was used most (434 out of 962 publications), followed by DMEM/F12 (230 out of 962 publications), MEM/F12 (68 out of 962 publications), DMEM high glucose (46 out of 962 publications), RPMI 1640 (37 out of 962 publications), Cosmedium-001 (21 out of 962 publications) and MEM (20 out of 962 publications) (Table 1). Furthermore, media used were supplemented with antibiotics/antimycotics (65.6% of the articles), glutamine (23.9% of the articles), non-essential amino acids (9.8% of the articles), sodium pyruvate (6.3% of the articles) or other components, such as HEPES, sodium carbonate, uridine or L-lysine, in different combinations (Table 1; for more detailed information of the media composition of each article, see Additional file 2). Careful choice of medium type and composition is crucial, e.g. the use of DMEM or RPMI changed the metabolome and the differentiation capacity of a number of cell lines [12, 13]. Evidence is now accumulating that nutrient availability, and also the degree of oxygenation, affects wiring through different metabolic pathways and that the intracellular levels of glutamine, alpha-ketoglutarate, pyruvate and NAD+/NADH redox ratio and concentration are determining factors in the epigenetic control of gene expression during differentiation, e.g. via effects on histone-lysine demethylases and DNA demethylases [14]. Furthermore, supplementing media with sodium pyruvate has been shown to be protective against oxidative stress [15, 16] and as such may affect the outcome of experiments that study the involvement of oxidative damage in PD. In 80% of the articles, the media described were supplemented with 10% fetal bovine serum, although some authors use other concentrations of serum, ranging from 5 to 20%, or serum from other species, such as horse (Table 1). Taking into account that serum includes growth factors, hormones, amino acids and lipids that can influence cell growth and differentiation, the use of different serum concentrations, serum from different species or even serum from different batches may influence the outcome [17]. It is of note here that variability in cell growth and sensitivity to various compounds depending on the media and substrate used for the growth of SH-SY5Y cells has already been reported [18].

**Table 1:** Compositions of the media used for culturing SH-SY5Y cells

Basal media		Supplements - serum		Supplements - others	
Name	#articles	Name	#articles	Name	#articles
DMEM	434	10% FBS	770	Antibiotics/antimycotics	631
DMEM/F12	230	15% FBS	70		
MEM/F12	68	5% FBS	33	Glutamine/GlutaMAX	230
DMEM (high glucose)	46	20% FBS	3		
RPMI 1640	37	None	2	NEAA	94
Cosmedium-001	21	Others	14	Sodium pyruvate	61
MEM	20	Unknown	70		
Other	36				
Unknown	70				

The table is divided into three parts (basal media, serum supplement and other supplements) and the number of papers involved is indicated (#articles). The Additional file 2 contains a more detailed description of all media and supplements used. Unknown refers to the articles that do not specify media composition. DMEM: Dulbecco’s Modified Eagle Medium; F12: nutrient mixture F12, MEM: Minimum Essential Media; FBS: fetal bovine serum; NEAA: non-essential amino acids

To appropriately acknowledge the various effects that differences in culture media composition can have on the cellular phenotype, it is thus imperative to systematically identify in future studies how various metabolic intermediates, ions, serum constituents and substrates influence aspects of growth and differentiation of SH-SY5Y cells. Only then the protocols for experiments with PD cell models can be widely standardized.

### Phenotype and differentiation of SH-SY5Y cells

The use of the SH-SY5Y cell line is not restricted to PD-research; this cell line has also been used in other areas of neuroscience, including research on Alzheimer’s disease, neurotoxicity, ischemia or Amyotrophic Lateral Sclerosis, among others [11, 19, 20]. To obtain derivative cells with a neuronal phenotype, multiple differentiation protocols have been described [21–25], but details about the final population of cells, regarding the fate-choices and fate-specification of cells that undergo terminal differentiation, have not been systematically reported. Since PD is characterized by the death of SN DAergic neurons, the degree to which this cell line displays a DAergic phenotype is a key aspect regarding the validity of the model. In this respect, 392 out of the 962 papers state that SH-SY5Y cells have a DAergic phenotype without actually showing supporting evidence. Only a few papers cite previous work showing the DAergic phenotype. Another large proportion of articles (432 out of 962) does not provide any statement about the DAergic phenotype or the rationale behind the choice of the cell line for use in PD-research. Among the remaining publications, in 76 papers the SH-SY5Y lineage is represented as a PD-model with DAergic properties or a toxin-induced PD-like phenotype, another 56 publications report the analysis of the DAergic phenotype, and in 7 papers cholinergic, neuronal or noradrenergic phenotypes are mentioned (Table 2).

**Table 2:** Papers reporting the DAergic phenotype of the SH-SY5Y cell line and techniques used

DAergic phenotype	#articles	#differentiated
Not stated	432	70
Stated and not checked	392	50
PD model/DAergic properties/toxin	76	12
Stated and checked^a^	48	22
Not stated but checked^a^	7	3
Others	7	2
Technique	Single	Multiple
ICC	11 (4/7)	15 (6/9)
WB	10 (6/4)	23 (13/10)
qPCR	1 (1/0)	13 (7/6)
DA uptake/content	2 (2/0)	6 (3/3)
Not shown	4 (3/1)	0

The top part of the table indicates the number of papers that do specify or do not specify the DAergic phenotype of the SH-SY5Y cell line and whether or not the authors checked the phenotype. “Others” refers to articles mentioning other neuronal linages, including cholinergic, neuronal and noradrenergic phenotypes. Shown are the total number of articles (#articles) and the number of articles in which forced differentiation was employed (#differentiated). The bottom part of the table summarizes the techniques used in the publications that checked the phenotype (^a^). Publications are divided into ‘single’ (i.e. papers that use only one method to check the DAergic phenotype) and ‘multiple’ (papers that use multiple complementary techniques). Between brackets: the number of studies that checked the DAergic phenotype in undifferentiated/differentiated cells. *ICC* immunocytochemistry, *WB* western blot, *qPCR* quantitative polymerase chain reaction, *DA* dopamine

**Fig. 2:**
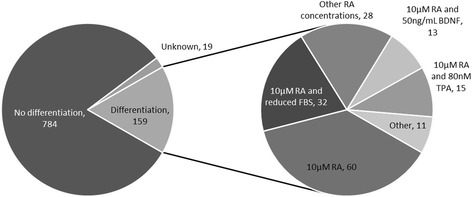
Papers reporting the differentiation of the SH-SY5Y cell line for PD-research. *Left*: Proportion of studies that do not use differentiation protocols (no differentiation), those that do not specify the differentiation status (unknown) and those that include a differentiation regime (differentiation). Among the papers in which differentiated cells were used, the main differentiation treatments used are depicted in the right chart, including 10 μM retinoic acid (RA), 10 μM RA and reduced fetal bovine serum (FBS), other concentrations of RA, 10 μM RA and 50 ng/ml brain-derived neurotrophic factor (BDNF) and 10 μM RA and 80nM 12-O-Tetradecanoylphorbol-13-acetate (TPA). Other includes 10 μM RA, 1%FBS and 0.3 mM dibutyryl-cAMP; 10 μM RA or 10 μg/mL BDNF; 10 μM RA and 80nM TPA or 50 ng/mL BDNF; 100 ng/mL of GDF5 or recombinant BMP2; neurobasal media with 6-10nM staurosporine or B27 supplement, 2 mM L-glutamine and 10 μM RA; 10 μM RA and 5 μM cAMP; 50 ng/ml GDNF; 10 μM RA and 80nM tissue plasminogen activator

The phenotype of SH-SY5Y cells can be manipulated by inducing different programs of terminal neural differentiation. However, in 81, 5% of the published studies no differentiation regime was used (Fig. 2), for which in only seven publications a reason was given. Among the studies that do report on forced differentiation, the most common method employed is the addition of retinoic acid (RA) in concentrations ranging from 5 μM to 100 μM, for a period of time from 24 hours to 21 days, and, sometimes, a reduction of the concentration of serum in the media (Fig. 2). It has been reported that RA treatment upregulates expression of neuronal and DAergic markers and increases susceptibility to DAergic neurotoxins [26]. However, other studies have observed increased neuronal markers upon RA differentiation, but no change in DAergic markers and decreased susceptibility to DAergic neurotoxins [27]. The phenotypic effect of RA on SH-SY5Y cells has been systematically studied, including the induction of a terminal neural phenotype with, specifically, a DAergic-like character [28]. Conversely, RA-mediated differentiation of SH-SY5Y cells has been associated with the induction of a cholinergic rather than DAergic phenotype [29]. Here it is important to note that RA has been found to partially protect SH-SY5Y cells against proteasome inhibitors [30]. In view of this finding, the results of studies examining proteasomal dysfunction and involving RA-differentiated SH-SY5Y cells as PD-model should be interpreted with care. The second method of choice to differentiate SH-SY5Y cells is a sequential treatment with RA, usually 10 μM, and 12-O-Tetradecanoylphorbol-13-acetate (TPA), mostly added in a concentration of 80nM (Fig. 2). This protocol has been demonstrated to differentiate SH-SY5Y cells more efficiently to DAergic-like neurons [31–33]. Early studies on the use of RA and TPA (alone or in combination) to differentiate SH-SY5Y cells have shown that these compounds induce various neuronal-like populations, with a strong increase of NA content when using only TPA [7]. In view of these differences, it is important to realize that a set of neurons each synthesizing a separate neurotransmitter (s) has a distinct transcriptional profile [34]. Even neurons synthesizing a specific neurotransmitter can be classified into several subpopulations, each with a clearly defined signaling function in a particular (brain) region and an explicit vulnerability for stress factors [35]. The third approach that is commonly used for differentiation induction involves the sequential treatment with RA, usually 10 μM, and 10-100 ng/mL of brain-derived neurotrophic factor (BDNF) (Fig. 2). This procedure leads to a homogeneous neuronal population with expression of neuronal markers and decreased proliferation [21]. The phenotypic outcome of this RA/BDNF differentiation protocol is, however, still somewhat controversial as it has been described as sympathetic cholinergic, based on evidence from target-directed qPCR and microarray studies which pointed into the direction of increased levels of acetylcholine transporter, choline acetyl transferase and neuropeptide Y [36, 37], but also as dopaminergic by others [38]. Moreover, inhibition of cell growth has not always been replicated when employing this procedure [24]. Additional protocols used for differentiation may involve combinations of the above-mentioned methods, or a combination of 10 μM RA and 0.3-5 mM dibutyryl cyclic adenosine monophosphate (dbcAMP) [39, 40], or of 10 μM RA for 3 days and 80nM tissue plasminogen activator [41] or the protocol was not specified. Differentiation may also be caused by 200 ng/mL growth/differentiation factor 5 (GDF5) [42], recombinant bone morphogenetic protein 2 (BMP2) [42], staurosporine [43, 44] or 50 ng/mL glial cell line-derived neurotrophic factor (GDNF) [45]. The pros and cons of the differentiation of the SH-SY5Y cell line to obtain a relevant model for PD have been reviewed more extensively elsewhere [46]. Again, proper characterization of the differentiated cells is crucial. Next to the more conventional “ensemble” approaches, such as western blotting, transcriptomics or proteomics, the use of novel single-cell microscopy and single-cell RNAseq approaches will become instrumental to provide more detailed phenotypic profiling of the differentiated cell population [47, 48].

In view of the relevance of the DAergic system in PD, it is striking that among the papers using SH-SY5Y cells in PD research only 55 out of 962 examine the DAergic phenotype of the cell line (Table 2; for a list and details of the articles that checked the DAergic phenotype see Additional file 3). Remarkably, in some cases the DAergic phenotype has been studied but the results were not shown. The methods used to determine the SH-SY5Y DAergic phenotype include immunocytochemistry (ICC), western blot (WB), quantitative polymerase chain reaction (qPCR) and DA uptake/content. Of note, “ensemble” methods like WB and qPCR do not allow a distinction between expression changes in the whole population or in just a subset of cells. In this respect, ICC represents a more reliable technique to check the phenotype of the cell population. ICC with DAergic markers has been used in 26 out of the 56 articles: 10 in undifferentiated and 16 in differentiated cells (in general compared with undifferentiated controls). Comparison of all the provided ICC images, taking into account the cell source, media composition and differentiation method (when applicable), did not allow us to draw definitive conclusions about the phenotype of the undifferentiated cells, or about differences in outcome between various differentiation methods. Of note, no or only a few positive control images are shown in most articles, hindering the comparative literature survey. Also, SH-SY5Y cells are known to respond inconsistently to the same differentiation treatment, depending on their cell source [49] or possibly passage number. Therefore, the variation reported may be at least in part due to the origin of the cells and different aspects regarding their handling, highlighting the importance of the proper reporting of all protocols involved.

### Mimicking PD

In order to create SH-SY5Y-derived cell models that mimic PD, strategies are used based on drug treatment and/or genetic approaches with the manipulation of expression of candidate genes that have emerged from genetic studies in PD-families. Most papers (800 out of 962) choose one pharmacological or genetic strategy to force manifestation of a PD-like phenotype, but also multiple variants or combinations of these strategies have been used. The most-used compounds in drug-based approaches are 1-methyl-4-phenylpyridinium (MPP+), 6-hydroxydopamine (6-OHDA) and rotenone, which dysregulate multiple cellular pathways, focusing on mitochondrial dysfunction and oxidative stress (Table 3). MPP+ is the toxic metabolite of 1-methyl-4-phenyl-1,2,3,6-tetrahydropyridine (MPTP), a by-product in the synthesis of 1-methyl-4-phenyl-4-propionoxy-piperidine (MPPP), a synthetic analog of heroin, which causes severe parkinsonism in humans when injected intravenously [50]. Since SH-SY5Y cells do not have the machinery to transform MPTP to MPP+, the metabolite itself is administered. Once inside the cell, MPP+ inhibits complex I of the electron transport chain, impairing mitochondrial respiration and increasing reactive oxygen species (ROS) production, and redistributes DA to the cytosol, where it is oxidized and generates more ROS (reviewed in [51]). 6-OHDA is a catecholaminergic neurotoxin and its molecular mechanisms of action have been reviewed elsewhere [52]. Briefly, 6-OHDA enters catecholaminergic neurons via DA or NA transporters; it accumulates inside of the cell and triggers the formation of ROS and catecholamine quinones, leading to oxidative stress and cell death. Furthermore, 6-OHDA may inhibit complexes I and IV of the electron transport chain [53], although this has not been confirmed by others [54]. Exposure to rotenone, a highly lipophilic insecticide, has been linked to sporadic PD [55] and can directly enter cells, independently from transporters, to inhibit complex I, impairing mitochondrial respiration and enhancing ROS production, and inhibit proteasomal activity (reviewed in [56]).

**Table 3:** Drug-based and genetic methods used to induce a PD-like phenotype in SH-SY5Y cells

PD-mimic			
	Single	Multiple	Total
MPP+	169	63	232
Manipulation of expression of familial genes	180	53	233
6-OHDA	141	47	188
Rotenone	69	56	125
Dopamine	33	26	59
H_2_O_2_	12	34	46
NM (R) Sal/Salsolinol	21	6	27
Paraquat	13	12	25
Lactacystin	8	11	19
Other treatments			112

Listed are the most commonly used treatments to mimic PD in the SH-SY5Y cell line as well as the number of articles that use one (single) or, to validate the results, more than one (multiple) treatment. Other treatments include conditioned media from glial cells, MG132, SIN-1, staurosporine, thapsigargin, carbonyl cyanide m-chlorophenyl hydrazine (CCCP), tunicamycin, epoxomicin, bafilomycin, neuromelanin, miRNAs, A-β1, BmK1, L-buthionine-(S, R)-sulfoximine (BSO), Conduritol B epoxide (CBE), Ciplastin and PSI. MPP+: 1-methyl-4-phenylpyridinium; 6-OHDA: 6-hydroxydopamine; H_2_O_2_: hydrogen peroxide

**Fig. 3:**
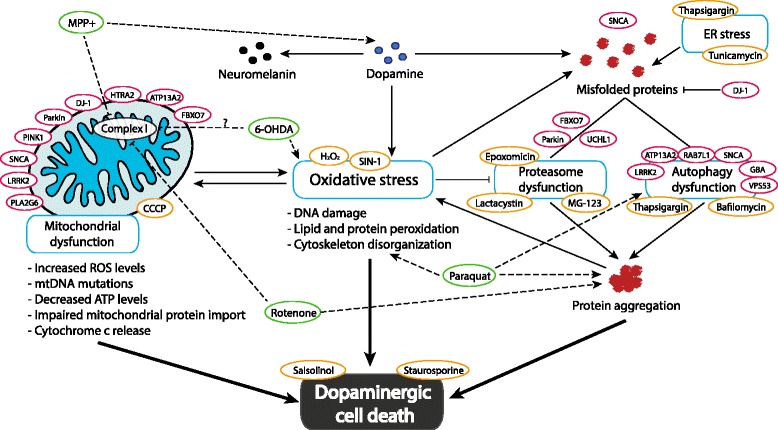
The molecular mechanisms dysregulated in PD and thought to lead to DAergic neuronal cell death. Up to now, 14 genes have been consistently associated with familial PD (*red circles*; [38]). The analysis of the functions of the corresponding (mutated) proteins and the resulting cellular abnormalities has allowed the identification of the depicted main pathways underlying PD: mitochondrial, proteasomal and autophagy dysfunction, protein aggregation, dopamine metabolism and oxidative stress, leading to DAergic cell death [39–44]. *Green circles*: compounds dysregulating multiple cellular processes linked to PD and used to mimic PD with dotted lines pointing towards their targets. *Orange circles*: drugs that specifically act on one of the processes are placed next to their targeted pathway

Next to drug-based strategies also genetic approaches (e.g. knock down or forced overexpression of genes with mutations found in familial cases of PD) have been widely used to induce a PD-like phenotype (Table 3). Altogether 19 loci segregating with familial forms of PD are now known [57]. Of these only a subset have been used for reverse genetic manipulation, and overexpression of genetically encoded mutated variants of α-synuclein (A30P, A53T, E46K, G51D, H50Q, S129A, S129D, S129E) or extracellularly added α-synuclein variants is by far the most commonly used method to mimic PD in SH-SY5Y. Functional and rescue studies involving knockdown, overexpression or mutated forms of other genes, such as LRRK2 (G2019S, I2020T, R1441C, Y1699C), PINK1 (G309D, P209A, P399L, T313M), DJ-1 (A39S, C53A, C106A, L166P), ATP13A2, PLA2G6, GBA and Parkin (C289G, C431F, G328E, G430D, K161N, R42P, T240N, T240R, R265C, W453stop), have also been performed. Gene knockdown was mostly performed by transfection with siRNA or shRNA, while stable expression of mutated genes was achieved by their insertion into vectors such as pcDNA3.1 and transfection with reagents like Lipofectamine® 2000 (ThermoFisher Scientific) or FuGENE ® (Promega), adenoviral infection or lentiviral transduction. Surprisingly, novel revolutionary technologies such as the use of CRISPR/Cas9 or TALEN have not yet been applied in studies on PD, but we anticipate that reports on these tools for precision gene-editing will appear soon. Collectively, the studies mimicking the genetic mutations present in PD have allowed the identification of a diverse set of pathways and molecular processes that are involved in the manifestation of the disease [58], including mitochondrial and mitophagy dysfunction [59, 60] and proteasomal and autophagy dysregulation, leading to protein aggregation [61–63]. One complementary strategy to study PD in cells is to interfere directly with one of these processes by administering specific compounds, with agonistic or antagonistic activity, such as hydrogen peroxide (oxidative stress), lactacystin/MG-123 (proteasome inhibitors), tunicamycin (N-glycosylation inhibitor, triggers ER stress), bafilomycin (inhibitor of vacuolar H^+^ ATPase, leading to autophagy dysfunction), thapsigargin (inhibitor of the sarco/endoplasmic reticulum Ca^2^^+^ ATPase, resulting in ER stress and autophagy inhibition), carbonyl cyanide m-chlorophenyl hydrazone (CCCP) (inhibitor of oxidative phosphorylation, leading to mitochondrial dysfunction), Conduritol B epoxide (CBE) (GBA inhibitor), or salsolinol/staurosporine (cell death). Intriguingly, staurosporine, a broad-spectrum kinase inhibitor, has been used in some PD-related publications to induce cell death [64–72], while other publications have used it to induce DAergic differentiation and study PD-related features [43, 44]. Early studies on SH-SY5Y cells showed differentiation towards a neuronal phenotype upon treatment with staurosporine [73, 74], which later has been characterized as catecholaminergic-like [75]. Therefore, the effects of different concentrations of staurosporine on SH-SY5Y cells should be characterized carefully to properly interpret studies that used this drug either as a differentiation agent or as an inducer of apoptosis. Figure 3 summarizes the now known cellular processes that are dysregulated in PD, based on the analysis of the functions of the proteins encoded by the (mutated) familial PD genes, and the use of PD-mimicking drugs in SH-SY5Y cells.

### Reproduction of PD-associated cellular phenotypes

Apart from the DAergic phenotype, the ability of the cell line to reproduce the cellular abnormalities of PD is crucial for the validity of the model. One of the main hallmarks of PD is α-synuclein aggregation [5]. To mimic this pathological feature, overexpression of WT α-synuclein or stable expression of one of its familial mutations, such as A53T or A30P, has been successfully used [76–78]. Nevertheless, these manipulations do not always inevitably lead to increased formation of inclusions. Therefore, triggers such as cell differentiation together with FeCl_2_ (and H_2_O_2_) treatment, or Hsp70 blockage, are sometimes needed to observe α-synuclein aggregation [79–82]. These different outcomes are possibly due to the specific α-synuclein mutation used [77] or the level of expression achieved by the various constructs [78]. Interestingly, spontaneous α-synuclein aggregation has been reported in non-transfected SH-SY5Y cells [83]. Moreover, both differentiated and undifferentiated SH-SY5Y cells are sensitive to extracellular α-synuclein-induced toxicity [84]. The cell line has also been used to study the kinetics and mechanisms of α-synuclein degradation [78, 85], the link between α-synuclein aggregation and intracellular calcium [86], and other pathological changes of α-synuclein, such as its carboxyl-terminal cleavage [32]. Other PD-related problems, such as abnormal mitochondrial function, oxidative stress and autophagy or proteasomal dysfunction, have been reproduced in SH-SY5Y cells as well. These hallmarks are usually triggered by the administration of specific drugs (Fig. 3) or, alternatively, by the knockdown of a gene corresponding to a familial PD-gene or expression of a familial PD-gene. For example, silencing of PINK1 leads to mitochondrial dysfunction [87, 88] and mutated forms of α-synuclein impair proteasomal activity [89]. The additive effects that are caused by co-expression of familial PD genes can also be observed in this cell line (e.g. cotransfection of α-synuclein and LRRK2 enhances the formation of aggregates, phosphorylation, cell-to-cell transmission and extracellular release of α-synuclein [90]). Therefore, SH-SY5Y cells represent an attractive tool to study most cellular alterations linked to PD and strategies to ameliorate their effects, but a careful experimental setting is required when analyzing α-synuclein aggregation, since the findings vary between studies. Yet, not all aspects of PD pathobiology can be faithfully studied in SH-SY5Y cells, and therefore investigations into for example electrophysiological abnormalities or neurochemical dysfunction require other and more complex models, such as primary dopaminergic cultures or brain slices, ex vivo.

### In vitro and in vivo models used in parallel with SH-SY5Y cells

PD is characterized by the loss of DAergic neurons from the SN [5]. Thus, primary cultures of neurons from this brain area of patients and controls may be considered the most reliable models to unravel the molecular mechanisms underlying this disease. However, the inaccessibility and lack of proliferation of such neurons largely precludes their use. Conversely, as discussed above, the use of a proliferative and more uniform model like the SH-SY5Y cell model has also limitations. Therefore in many studies other cell types have been employed in parallel. Of the articles analyzed, 67.6% reported experiments performed exclusively in the SH-SY5Y cell line and 19.7% used in parallel other cell lines with a neuronal phenotype, including rodent mesencephalic primary cultures and (mainly cortical) primary neurons, stem cells, PC12 cell line, Neuro-2a cell line or MN9D cell line (Table 4). The remaining 12.7% used cell lines that are not neuronal (−like), such as HEK293, HeLa or glial cells. Primary cell cultures are physiologically more relevant than immortalized cell lines, but they are difficult to maintain and, depending on the age of the source animals or the dissection accuracy, can introduce experimental variability [91]. Since the SN is located in the mesencephalon, or midbrain, rodent mesencephalic primary cultures are enriched for the cell population of interest, the SN DAergic neurons. Primary cultures from other brain regions (mainly cortex) have also been extensively used as complementary in vitro models, but these cultures consist of mixed populations of various neuronal subtypes. Furthermore, primary cultures of DAergic neurons from other species, such as the worm *C. elegans*, have been used [92, 93]. In general, the overtly less physiologically relevant permanently established cell lines are easier to maintain than cells in primary cultures (because the cell lines are generally rendered immortal) and can sometimes be differentiated into terminal neuronal populations. The PC12 cell line (ATCC® CRL-1721™) has been derived from a rat pheochromocytoma and widely used to study PD. This lineage has a chromaffin-like character, and shares its embryonic origin with DAergic neurons. Nerve growth factor treatment of PC12 cells induces differentiation into a catecholaminergic-like phenotype [94, 95]. The Neuro-2a cell line (ATCC® CCL-131™) has been derived from a mouse brain neuroblastoma and can be differentiated into neuronal-like cells [96] and, more specifically, to DAergic neurons by a dbcAMP treatment [97]. Other PD cellular models include cell hybrids, such as the MN9D cell line [98], and derivatives from the neuroblastoma SK cell line. A full list of neuronal (−like) cell models used in parallel with SH-SY5Y cells can be found in Additional file 4. It is important to note that most of the alternative lineages are derived from species other than human. Finally, patient-derived induced pluripotent stem cells (iPSCs) that can be differentiated into DAergic neurons (and possibly other relevant cell types such as glial cells) represent an emerging new category of cell models for PD [99–101].

**Table 4:** Use of alternative cellular models in parallel to SH-SY5Y cells

Alternative cellular models	#articles
No other neuronal cell lines	772
Other neuronal (−like) cell lines	190
Mesencephalic cultures (mouse/rat)	54
Primary neurons (cortical mainly) (mouse/rat)	54
PC12 (rat)	39
Neuro-2a (mouse)	13
hESC, NPSC, hMSC, iPSCs (human)	9
SK-N-BE (2)-M17(M17) (human)	9
MN9D (mouse)	7
Other	45

The table specifies the number of articles (#articles) that do not use any neuronal cell line other than SH-SY5Y cells; and those that do use another neuronal (−like) cell line (and the most commonly used ones). Sometimes an article uses multiple alternative cell lines and, thus, the addition of the individual values of other neuronal (−like) cell lines is larger than the number of articles that use other neuronal (−like) cell lines (190). More detailed information on these other cell lines can be found in Additional file 4

Apart from cellular models, about 21% of the articles that use SH-SY5Y cells also employ an animal model for PD to validate and further understand the cellular results. The animal models include mouse, rat, *C. elegans* and fruit fly and the PD features in these in vivo models are mimicked with MPTP, 6-OHDA, rotenone or genetic mutations (reviewed in [102, 103]).

## Conclusions

This systematic review illustrates the “popularity” and broad use of the neuroblastoma cell line SH-SY5Y in PD research and underlines some of its drawbacks. SH-SY5Y cells have been used to study the molecular and cellular mechanisms underlying the effects of some of the PD-related toxins, to perform functional studies on familial PD genes, and to test putative protective compounds for PD treatment. Thus, this cell line has been a valuable asset to help unravel the molecular complexity of PD. However, SH-SY5Y cells are not purely DAergic because the cell line was obtained as a neuroblastoma derivative and thus has cancerous properties that influence its differentiation fate, viability, growth performance, metabolic properties and genomic stability. Hence, SH-SY5Y cells possess physiological characteristics which differ greatly from the normal DAergic neuronal features. Reports on the exact SH-SY5Y phenotype are contradictory. Differences in cell source and maintenance in culture, perhaps of epigenetic character, could explain these variations, but the lack of accurate reporting of experimental protocol parameters and inaccurate listing of individual characteristics of cell lineages kept at different laboratories hinders the drawing of firm conclusions. Therefore, the cell source has to be specifically indicated and more studies on the effects of media composition on the cell population are needed to compare findings, and catalyze reproducibility and progress with this PD-model. In addition, the use of other neuronal (−like) cell lines, such as those reviewed here, and animal models in parallel with SH-SY5Y cells may help to validate the findings. The choice of these additional models should take into account aspects such as species differences, tumorigenic properties and time and resource requirements. A further topic regarding the use of SH-SY5Y cells concerns the differentiation regime that - until now - has been used to drive the cell line towards a DAergic phenotype. Variations in the outcome of the differentiation protocol could again be due to the origin and handling of the cells. Furthermore, the use of chemical compounds to differentiate the cell line into a more DAergic or neuronal population may affect parameters that are not directly linked to the desired phenotype, and they may produce confounding effects. The systematic use of ICC and other single cell assays, together with qPCR and WB to characterize the phenotype of the entire cell population, is required for a proper validation of the DAergic phenotype of the SH-SY5Y cells as a disease model. The method of choice to model PD is crucial especially because the onset of this multifactorial disease involves both genetic and environmental factors. Genetic as well as chemical approaches have been used in the functional studies on SH-SY5Y cells to target one or multiple pathways linked to PD. In any case, the use of multiple approaches in parallel is recommended and expected to be facilitated by current developments in the fields of chemical biology and reverse-genetics (i.e. CRISPR/Cas9 applications) that will allow a much broader application of chemical libraries for cell-signaling inhibition and genome editing, respectively. These novel opportunities together with the proper exploitation of the already well-established procedures for cell culturing will allow the standardization of the use of the SH-SY5Y cell line and maximize the benefit from this appealing cell model for PD.

## Additional files

Additional file 1: List of articles resulting from the literature search and article inclusion/exclusion in this review. Complete list of articles that were obtained with the search terms “Parkinson”, “Parkinson’s”, or “Parkinson’s disease” and “neuroblastoma”, “SH-SY5Y”, “SHSY5Y, or “SHSY-5Y” in PubMed until the 22^nd^ of November 2016. The inclusion and exclusion criteria are specified. (XLSX 263 kb)
Additional file 2: Compositions of the media used for culturing SH-SY5Y cells. The table displays the composition of the media used in each of the articles included in the review. (XLSX 55 kb)
Additional file 3: Techniques and markers used to determine the DAergic phenotype of SH-SY5Y cells. List of articles in which the DAergic phenotype of the cell line was experimentally validated, and the techniques and markers that were used, together with the cell source, media used and differentiation included or not. (XLSX 18 kb)
Additional file 4: Alternative neuronal (−like) cell lines used to validate the findings in SH-SY5Y cells. The table contains the neuronal (−like) cell lines that have been used in parallel with SH-SY5Y cells to study PD and the species from which they are derived, the cell type and the number of articles that used the cell line. (XLSX 13 kb)

## Abbreviations

6-OHDA: 6-hydroxydopamine
ATCC: American Type Culture Collection
BDNF: Brain-derived neurotrophic factor
DA: Dopamine
DAergic: Dopaminergic
DSMZ: German collection of Microorganisms and Cell Cultures
ECACC: European Collection of Authenticated Cell Cultures
ICC: Immunocytochemistry
MPP+: 1-methyl-4-phenylpyridinium
MPTP: 1-methyl-4-phenyl-1,2,3,6-tetrahydropyridine
NA: Noradrenaline
PD: Parkinson’s disease
qPCR: Quantitative polymerase chain reaction
RA: Retinoic acid
ROS: Reactive oxygen species
SN: Substantia nigra
TPA: 12-O-Tetradecanoylphorbol-13-acetate
WB: Western blot
